# Syndrome du canal carpien bilatéral modéré amélioré par la mobilisation neurodynamique du nerf médian: à propos d'un cas

**DOI:** 10.11604/pamj.2022.42.231.33240

**Published:** 2022-07-26

**Authors:** Hassan Beddaa, Bouchra Kably, Basma Marzouk, Youness Azemmour, Ismail Bouzekraoui Alaoui, Nazha Birouk

**Affiliations:** 1 Laboratoire de Biostatistiques de Recherche Clinique et épidémiologique, Université Mohamed V de Rabat, Rabat, Maroc,; 2 Service de Neurophysiologie Clinique, Hôpital de Spécialités, Centre Hospitalier Universitaire, Université Mohamed V de Rabat, Rabat, Maroc,; 3 Comité National Olympique Marocain, Rabat, Maroc

**Keywords:** Douleur, electroneuromyogramme, techniques de mobilisation neurodynamique, syndrome de canal carpien, cas clinique, Pain, electroneuromyogram, neurodynamic mobilization techniques, carpal tunnel syndrome, case report

## Abstract

Le syndrome du canal carpien est la neuropathie canalaire la plus fréquente affectant le membre supérieur. Plusieurs approches thérapeutiques sont utilisées pour traiter ce syndrome, dont le traitement conservateur souvent de première ligne. Nous rapportons l´observation d´une patiente de 61 ans, qui s´est présentée au service de neurophysiologie clinique de l´hôpital de spécialités de Rabat pour un syndrome du canal carpien sensitif modéré et bilatéral confirmé par électroneuromyogramme (ENMG). L´intervention a consisté en une thérapie manuelle incluant une mobilisation neurodynamique du nerf médian en bilatéral. L´évolution a été marquée par une disparition de l´engourdissement nocturne et l´ENMG de contrôle a montré une nette amélioration des paramètres de conduction nerveuse. D´après ce résultat positif, la mobilisation neurodynamique du nerf médian pourrait être considérée comme une piste envisageable dans le cadre du traitement conservateur du syndrome du canal carpien.

## Abstract (en)

Carpal tunnel syndrome is the most common neuropathy affecting the upper limb. Several therapeutic approaches are used to treat this syndrome, including conservative treatment, often used as the first line treatment. We here report the case of a 61-year-old female patient, presenting to the Department of Clinical Neurophysiology of the Specialty Hospital, Rabat, with moderate and bilateral carpal tunnel syndrome with sensory loss confirmed by electroneuromyography (ENMG). Manual therapy, including bilateral median nerve neurodynamic mobilization, was performed. Patient´s outcome was marked by disappearance of nocturnal numbness and follow up ENMG showed a marked improvement in nerve conduction parameters. Based on this positive result, neurodynamic mobilization of the median nerve may be considered as a possible approach for the conservative treatment of carpal tunnel syndrome.

## Introduction

Le syndrome du canal carpien (SCC) correspond à une compression du nerf médian au niveau du poignet. Il est considéré comme la neuropathie canalaire la plus fréquente touchant le membre supérieur et pouvant affecter significativement la qualité de vie des patients. A un stade précoce le traitement conservateur est largement proposé pour traiter cette affection [1]. Il comprend entre autres la mobilisation neurodynamique et en particulier des techniques de glissement du nerf médian. En effet, plusieurs études antérieures ont montré son effet positif sur la douleur et la fonction du membre supérieur chez les patients souffrants de ce syndrome. Toutefois, les études évaluant l´effet de ces techniques sur la conduction nerveuse du nerf médian sont rares [2]. Dans cet article nous allons présenter et discuter une observation concernant l´évolution des signes cliniques et des résultats de l´ENMG après 20 séances de thérapie manuelle incluant une mobilisation neurodynamique du nerf médian chez une patiente avec un SCC bilatéral modéré.

## Patient et observation

**Informations de la patiente**: il s´agit d´une femme âgée de 61 ans, médecin ophtalmologue de profession, sans antécédents médico-chirurgicaux qui s´est présentée au service de neurophysiologie clinique à l´hôpital de spécialités de Rabat pour un engourdissement intermittent des doigts à prédominance nocturne sans autres signes associés. L´interrogatoire a révélé l´impact psychologique et les inquiétudes de la patiente quant à l´évolution de son affection. Elle rapporte être gênée dans l´exercice de son métier et craignait que celui-ci ne soit impacté négativement du moment qu´il dépend amplement de ses prises manuelles fines et de coordination gestuelle.

**Résultats cliniques**: l´examen médical de cette patiente s´est révélé normal en dehors d´un signe de Tinel positif bilatéralement. L´examen n´a pas objectivé de limitation de la mobilité passive des deux membres supérieurs et du rachis cervical. L´évaluation de la douleur a été réalisée avec l´Echelle Numérique (EN) et ne trouvait pas de douleur spontanée. Le test de Spurling a été effectué pour écarter une névralgie cervico-brachiale dont les symptômes peuvent ressembler à ceux du SCC, il s´est avéré négatif [3]. Finalement, l´ULNT1 (*Upper Limb Neurodynamic Test 1)* a été réalisé en bilatéral pour mettre en évidence une mécano-sensibilité des deux nerfs médians [4] et s´est avéré positif à droite à -30° d´extension du coude et à gauche à -20° avec accentuation des symptômes bilatéralement par la manœuvre de différentiation structurelle (inclinaison controlatérale de la tête).

**Chronologie**: les symptômes de la patiente ont commencé depuis plus d´une année. Après avoir consulté plusieurs spécialistes et essayé plusieurs traitements médicamenteux, les symptômes n´ont pas régressés de manière significative. Conséquemment, la patiente a cumulé plusieurs arrêts de travail.

**Démarche diagnostique**: l´examen ENMG a montré une conduction nerveuse motrice normale, en particulier des latences distales motrices des nerfs médians dans les limites normales. On ne notait pas de différence significative entre les latences distales motrices entre les nerfs médian et ulnaire. L´examen a par contre objectivé une diminution de la vitesse de conduction sensitive de traversée du canal carpien pour le nerf médian droit à la stimulation de la paume et du troisième doigt et à la stimulation de la paume pour le nerf médian gauche concluant ainsi à une atteinte bilatérale modérée sensitive du nerf médian prédominant à droite.

**Intervention thérapeutique**: la patiente a été adressée par son médecin traitant pour 20 séances de rééducation à raison de 2 séances par semaine. L´intervention thérapeutique a consisté en un traitement des interfaces articulaires et musculaires du nerf médian en bilatéral, une mobilisation neurodynamique en glissement et en tension des deux nerfs médians en plus des exercices à reproduire régulièrement à domicile. Pour le traitement des interfaces mécaniques du nerf médian, une mobilisation de postériorisation de la tête humérale ainsi qu´une mobilisation des os carpiens ont été réalisées systématiquement, bilatéralement et passivement par le thérapeute. Dans un deuxième temps, le traitement des interfaces musculaires a consisté en une levée de tension des muscles petits pectoraux, sous-claviers et ronds pronateurs bilatéralement. Le traitement de ces interfaces mécaniques a visé dans un premier temps de permettre au nerf d´avoir un environnement favorable pour son glissement.

Dans un deuxième temps, il a été procédé à la mobilisation neurodynamique en glissement du nerf médian en bilatéral. Concrètement, la patiente a été placée en décubitus dorsal sur la table de traitement, le thérapeute du côté à traiter maintient le bras en abduction à 110°, en rotation externe à 90°, l´avant-bras en supination, le poignet et les doigts en extension avec le coude fléchi à 90°. La mobilisation consiste à étendre le coude jusqu´à l´extension complète possible en même temps de fléchir le poignet ce qui cause un glissement proximal du nerf médian et d´inverser la situation en fléchissant le coude en même temps d´étendre le poignet ce qui cause un glissement distal du nerf médian. C´est la mobilisation en glissement du nerf médian entre le coude et le poignet. Vers la 15^e^ séance, nous avons procédé à la mobilisation en tension du nerf médian en maintenant la même position sus-citée pour la description de la technique du glissement avec le coude maintenu toujours en extension et en mobilisant juste le poignet de flexion à extension. Cette dernière technique a visé de gagner sur l´élasticité du nerf médian dans la limite de mécano-sensibilité tolérée par la patiente. Pour les techniques de mobilisation neurodynamique, 3 séries de 60 secondes chacune ont été réalisées entrecoupées d´un temps de repos de 2 minutes entre elles. Finalement, des exercices d´auto-étirement du muscle petit pectoral et une pression ischémique sur le rond pronateur ont été appris à la patiente en plus des exercices de glissement du nerf médian. Elle a été invitée à les reproduire deux fois par jour chez elle à domicile.

**Suivi et résultats des interventions thérapeutiques**: à la fin des séances prescrites, la patiente a été évaluée concernant la douleur (0 sur l´EN), la mécano-sensibilité du nerf médian bilatéralement (pas de reproduction de symptômes avec l´ULNT1 et l´inclinaison controlatérale de la tête bilatéralement) et une satisfaction quant à la disparition de l´engourdissement nocturne des doigts. Le Tableau 1 résume l´examen clinique avant et après l´intervention. L´examen ENMG de contrôle a montré une très nette amélioration des paramètres de conduction des nerfs médians aussi bien des différences de latence motrices entre nerfs médian et ulnaire (avec recueil sur les muscles premier lombrical et 2^e^ interosseux) qui ont nettement diminué, que la normalisation des vitesses de conduction sensitive à la stimulation de la paume et du 3^e^ doigt. Seule persistait une discrète différence de latence distale sensitive entre nerf médian et ulnaire au 4^e^ doigt à droite, cette différence était normale à gauche. Les Tableau 2 et Tableau 3 montrent les principaux paramètres ENMG avant et après traitement.

**Tableau 1:** les tests cliniques réalisés avant et après le traitement

Variables	Côté droit		Côté gauche	
Test	Avant traitement	Après traitement	Avant traitement	Après traitement
**Phalen**	Négatif	Négatif	Négatif	Négatif
**Tinel**	Positif	Négatif	Positif	Négatif
**Spurling**	Négatif	Négatif	Négatif	Négatif
**ULNT1**	Positif (- 30°)*	Négatif	Positif(- 20°)*	Négatif

* Le test était considéré positif s´il reproduit les symptômes de la patiente et que ces symptômes changent avec la différentiation structurelle réalisée par inclinaison controlatérale de la tête. La mesure en degrés a été réalisée par goniomètre et concerne l´extension du coude.

**Tableau 2:** résultats de l´ENMG des deux nerfs médians avant et après le traitement

Variables	Conduction nerveuse motrice avant et après traitement							
	LDM (ms)				Amplitude poignet (mV)			
	Médian droit		Médian gauche		Médian droit		Médian gauche	
Avant traitement	3.44		2.97		8.9		9.5	
Après traitement	3.07		2.60		9.1		7.9	
Différence	(-)0.37		(-)0.37		(+)0.2		(-)1.6	
	**Conduction nerveuse sensitive avant et après traitement**							
	**Médian Droit**		**Médian Droit**		**Médian Gauche**		**Médian Gauche**	
	**VCS(m/s)**		**Amplitude (uV)**		**VCS (m/s)**		**Amplitude (uV)**	
	Doigt 3	Paume	Doigt 3	Paume	Doigt 3	Paume	Doigt 3	Paume
Avant traitement	37	32	21.5	95	23.8	43	144.2	38
Après traitement	44	40	20,2	137.2	22	50	145.1	45
Différence	(+)7	(+)8	(-)1.3	(+)42.2	(-)1.8	(+)7	(+)0.9	(+)7

ENMG: Electroneuromyogramme; LDM: Latence Distale Motrice; VCS: vitesse de conduction sensitive

**Tableau 3:** différence de latence motrice lombrical/IOD avant et après traitement(ms)

Variables	Main droite	Main gauche
Avant traitement	0.52	0.36
Après traitement	0.16	0.0
Différence	(-)0.36	(-)0.36

**IOD:** interosseux dorsaux

**Perspectives du patient**: la patiente a exprimé sa satisfaction concernant sa prise en charge et les résultats obtenus.

**Consentement éclairé**: un consentement éclairé a été donné par la patiente pour publier le cas.

## Discussion

En plus d´être considéré comme la neuropathie la plus fréquente touchant le membre supérieur et en particulier les travailleurs manuels [5], le SCC non traité peut causer des dommages permanents au nerf médian pouvant entrainer une altération de la fonction de la main [1]. Nous avons rapporté le cas d´une femme de 61 ans avec un SCC sensitif bilatéral modéré qui a été adressée en rééducation pour 20 séances à raison de 2 séances par semaine. La littérature rapporte en effet que le SCC affecte plus les femmes avec une présentation souvent bilatérale [6,7]. L´intervention a été réalisée par mobilisation neurodynamique en glissement puis progressivement en tension du nerf médian en plus du traitement de ses interfaces et des exercices à domicile. Les résultats ont montré une amélioration clinique (Tinel négatif, mécano-sensibilité améliorée, disparition de l´engourdissement nocturne) ainsi qu´une amélioration nette concernant les résultats de l´ENMG de contrôle et une satisfaction de la patiente. En effet, des études antérieures ont montré que l´amélioration de la mécanique du nerf influence positivement sa physiologie ce qui peut expliquer les résultats obtenus [8]. Dans le même sens, l´étude de Wolny et al en 2018 a conclu que la thérapie manuelle incluant des exercices neurodynamiques du nerf médian améliore l´état de santé général des patients atteints de SCC léger ou modéré.

Par ailleurs, il existe des contre-indications à la mobilisation neurodynamique telle qu´une suture nerveuse de moins de trois semaines, une hydrocéphalie non stabilisée ou une pathologie tumorale ou infectieuse du système nerveux [9]. Notre patiente ne présentait aucune contre-indication à la suite de l´examen médical. La majorité des études évaluant l´efficacité de la mobilisation neurodynamique en cas de SCC ont concerné les formes discrètes et modérées de ce syndrome et ont montré des résultats satisfaisants sur plusieurs dimensions et notamment la douleur et les activités de tous les jours [2,10] ce qui était le cas de notre patiente. La mobilisation neurodynamique pourrait constituer alors un choix thérapeutique prometteur surtout si elle est combinée à un traitement conservateur [11,12].

## Conclusion

L´introduction de la thérapie manuelle incluant une mobilisation neurodynamique du nerf médian en bilatéral a donné de bons résultats cliniques et a amélioré la fonction de conduction du nerf pour une patiente avec SCC bilatéral modéré. Le recours au traitement conservateur incluant la mobilisation neurodynamique pourrait donc améliorer l´état de santé des patients avec SCC.

## References

[1] Brininger TL, Rogers JC, Holm MB, Baker NA, Li ZM, Goitz RJ (2007). Efficacy of a fabricated customized splint and tendon and nerve gliding exercises for the treatment of carpal tunnel syndrome: a randomized controlled trial. Arch Phys Med Rehabil.

[2] Wolny T, Linek P (2018). The effect of manual therapy including neurodynamic techniques on the overall health status of people with carpal tunnel syndrome: a randomized controlled trial. J Manipulative Physiol Ther.

[3] Wainner RS, Fritz JM, Irrgang JJ, Boninger ML, Delitto A, Allison S (2003). Reliability and diagnostic accuracy of the clinical examination and patient self-report measures for cervical radiculopathy. Spine (Phila Pa 1976).

[4] Dilley A, Lynn B, See JP (2005). Pressure and stretch mechanosensitivity of peripheral nerve fibres following local inflammation of the nerve trunk. Pain.

[5] Dale AM, Harris-Adamson C, Rempel D, Gerr F, Hegmann K, Silverstein B (2013). Prevalence and incidence of carpal tunnel syndrome in US working populations: pooled analysis of six prospective studies. Scand J Work Environ Heal.

[6] Mondelli M, Giannini F, Giacchi M (2002). Carpal tunnel syndrome incidence in a general population. Neurology.

[7] Bagatur AE, Zorer G (2001). The carpal tunnel syndrome is a bilateral disorder. J Bone Jt Surg-Ser B.

[8] Shacklock M (1995). Neurodynamics. Physiotherapy.

[9] Pommerol P, Pommerol C (2013). Ostéopathie et thérapie manuelle du tissus neuro-méningée. SAURAMPS MEDICA.

[10] Wolny T, Saulicz E, Linek P, Shacklock M, Myśliwiec A (2017). Efficacy of manual therapy including neurodynamic techniques for the treatment of carpal tunnel syndrome: a randomized controlled trial. J Manipulative Physiol Ther.

[11] Kim S-D (2015). Efficacy of tendon and nerve gliding exercises for carpal tunnel syndrome: a systematic review of randomized controlled trials. J Phys Ther Sci.

[12] Ballestero-Pérez R, Plaza-Manzano G, Urraca-Gesto A, Romo-Romo F, Atín-Arratibel M de los Á Pecos-Martín D (2017). Effectiveness of nerve gliding exercises on carpal tunnel syndrome: a systematic review. J Manipulative Physiol Ther.

